# Achieving environmental sustainability via an integrated shampoo optimized BiLSTM-Transformer model for enhanced time-series forecasting

**DOI:** 10.1038/s41598-025-11301-6

**Published:** 2025-07-23

**Authors:** Asmaa Mohamed El-saieed, Nada A. Dief

**Affiliations:** 1 Department of Communication and Electronics, Mansoura High Institute of Engineering and Technology, Mansoura, Egypt; 2 Computers and Control Systems Department, Faculty of Engineering, Mansoura University, Mansoura, Egypt

**Keywords:** Deep learning, Renewable energy forecasting, Weather data prediction, BiLSTM, Transformer model, Energy science and technology, Renewable energy

## Abstract

Accurate forecasting plays a vital role in enhancing the efficiency of power systems, ensuring better resource management, and supporting strategic decision-making. This work presents BiLSTM-Transformer, a hybrid deep learning model that integrates Bidirectional Long Short-Term Memory (BiLSTM) networks with Transformer architecture to improve predictive performance in complex time-series tasks. The model employs a second-order optimization approach using Shampoo, which strengthens convergence stability and promotes better generalization during training. By effectively modeling both short-term variations and long-range dependencies in meteorological data, BiLSTM-Transformer achieves superior forecast accuracy across multiple evaluation benchmarks. The results highlight its potential as a reliable tool for supporting sustainable energy planning and smart grid operations.

## Introduction

Accurate forecasting of wind energy has been recognized as essential for the seamless integration of renewable resources into contemporary power systems. The intermittent nature of wind power has continued to pose challenges in terms of grid reliability, market efficiency, and operational stability. To mitigate such challenges, forecasting models have been developed to support resource scheduling and power dispatch, ultimately contributing to reduced operational costs and improved grid performance. Various approaches ranging from statistical models and hybrid techniques to Artificial Neural Networks (ANNs) and Numerical Weather Prediction (NWP) have been investigated to reduce the unpredictability associated with wind generation^1,2^.

Significant improvements in forecasting outcomes have been observed in diverse applications. In Tasmania, adaptive neuro fuzzy inference systems were shown to deliver superior performance in very short-term wind prediction, particularly in the estimation of wind vectors^3^. Additionally, data-driven ensemble approaches have been found to outperform single algorithm methods in short-term forecasting accuracy^4^. Advances in probabilistic modeling, such as the incorporation of circular variables and skewt distributions, have further contributed to the improvement of predictive models under complex meteorological conditions^5,6^.

The need for models capable of addressing the nonlinear behavior and temporal variability of renewable energy sources has led to the exploration of more sophisticated techniques. Although traditional time-series models such as ARIMA, ETS, and linear regression remain widely used due to their interpretability and simplicity^7^, they have often been limited by their inability to capture long-term dependencies and nonlinear relationships within weather data^8^. Consequently, deep learning models have been introduced to overcome these limitations. LSTM networks^9,10^, Transformers^11^, and other neural architectures^12^ have demonstrated considerable effectiveness in identifying both short- and long-range dependencies in sequential datasets^13^.

Despite these advancements, training stability and convergence efficiency continue to influence the reliability of deep learning models in forecasting applications. In this context, second-order optimization methods have been explored to improve learning robustness. Among them, Shampoo has been recognized for its ability to improve convergence and generalization through the use of Hessian-based preconditioning^9^. Although its computational demands are non-trivial, Shampoo’s effectiveness in stabilizing weight updates has made it a promising tool in domains involving complex temporal data.

In response to these challenges, a hybrid deep learning framework referred to as BiLSTMTransformer has been proposed in this study. This architecture has been constructed by integrating Bidirectional Long Short-Term Memory (BiLSTM) layers, which capture short-term variations, with Transformer layers, designed to model extended temporal dependencies. To further improve training outcomes, a layer specific optimization strategy utilizing Shampoo has been employed. This design has been tailored to align optimization techniques with architectural characteristics, thereby enhancing model performance.

The aim of this work is to present a robust and scalable forecasting solution that addresses the increasing complexity of renewable energy generation. The proposed BiLSTMTransformer model is evaluated using long-term weather data from multiple Egyptian locations. Its performance is assessed based on forecasting accuracy, training stability, and generalization ability. It is anticipated that the findings will contribute to the advancement of intelligent energy forecasting tools, ultimately supporting infrastructure planning and the global transition toward sustainable energy systems.

## Literature review and related works

Time-series forecasting has long been regarded an essential tool for recognizing patterns and trends in various domains, including energy, finance, and meteorology. Early forecasting models were capable of capturing simple linear relationships; however, as data structures grew increasingly complex, traditional methods such as ARIMA and ETS began to fall short in addressing nonlinearities and long-range dependencies in time-series data^14^. To overcome these limitations, more advanced forecasting models have been introduced.

ARIMA has served as a foundational model for time-series analysis, utilizing autoregression, differencing, and moving averages to model stationary data, where statistical properties remain constant over time. Although ARIMA has proven effective in modeling linear and seasonal patterns, its applicability diminishes in the presence of non-stationary or nonlinear dynamics, as often encountered in meteorological forecasting^15^. ETS, on the other hand, has been effective in modeling data with clear seasonal structures by decomposing series into error, trend, and seasonal components. However, its performance remains constrained when tasked with capturing nonlinear interactions or extended temporal dependencies^13^. Despite these drawbacks, both ARIMA and ETS continue to be employed due to their simplicity and interpretability, particularly in domains such as finance and climate modeling.

Linear regression has also been widely adopted as a baseline model for forecasting tasks due to its straightforward interpretability and computational efficiency^16^. However, its reliance on the assumption of linear relationships limits its effectiveness in real world datasets, especially those containing complex patterns, such as in weather data^17^. Although valuable for benchmarking, linear models are insufficient when capturing intricate temporal and nonlinear dynamics.

In response to these shortcomings, several neural network based approaches have been developed, including Gated Recurrent Units (GRUs), Convolutional Neural Networks (CNNs), Long Short-Term Memory networks (LSTMs), and Transformers. GRUs, a simplified variant of LSTMs, have been constructed by merging the input and forget gates into a single update gate, allowing for faster training and reduced computational overhead. While GRUs perform well on small datasets, their ability to capture long-range dependencies remains limited^18^.

CNNs, originally designed for spatial feature extraction in image processing, have been successfully repurposed for time-series forecasting due to their capacity to detect localized patterns. When combined with recurrent layers such as LSTM or GRU, CNN based architectures can identify short-term dependencies effectively. However, their limitations become apparent when modeling long sequences, especially in datasets characterized by high temporal variability^19^.

LSTM networks were introduced to resolve the vanishing gradient issue in traditional RNNs. By incorporating gated mechanisms that selectively retain and forget information, LSTMs have proven to be highly effective in capturing complex temporal dependencies over extended time horizons. These capabilities have rendered them particularly suitable for weather forecasting and other domains characterized by seasonal or irregular patterns^10,20^. Nevertheless, LSTMs can be computationally expensive and are sensitive to hyperparameter configurations, particularly when applied to lengthy input sequences^21^.

Transformers, first introduced in the context of natural language processing, have shown considerable promise in time-series applications due to their self attention mechanisms, which allow for efficient modeling of long range dependencies without reliance on recurrence^11^. By processing entire sequences in parallel, Transformers overcome some of the scalability limitations found in RNN-based models. However, challenges persist in capturing short-term dynamics, particularly when data is noisy or when datasets are relatively small.

To address the individual limitations of LSTMs and Transformers, hybrid models have been proposed. These models aim to combine the sequential pattern recognition capabilities of LSTMs with the global context modeling offered by Transformers. Studies have demonstrated that such hybrid architectures improve forecasting accuracy, especially in applications like weather prediction where both short and long-term dependencies must be captured simultaneously^22^.

Although traditional statistical models remain relevant for foundational forecasting tasks, the introduction of deep learning particularly LSTMs and Transformers, has significantly advanced the field’s ability to model complex and nonlinear temporal structures. Hybrid architectures that leverage the strengths of both paradigms have emerged as powerful tools for improving the reliability and precision of forecasts in dynamic environments.

The optimization strategy underlying these models is also a determining factor in their performance. Shampoo, a second-order optimizer, has been shown to improve convergence stability and predictive accuracy through Hessian-based matrix preconditioning^23^. By allowing gradient updates to be more precisely scaled, Shampoo contributes to better generalization, especially when applied to high-dimensional and temporally complex datasets. Despite its increased computational demands, it has demonstrated advantages over more commonly used first-order methods, particularly in applications requiring robust learning behavior over extended training cycles. As illustrated in Table 1, traditional models such as ARIMA, ETS, and Linear Regression offer high interpretability and computational efficiency but fall short in handling nonlinear patterns and long-term dependencies. In contrast, deep learning models particularly LSTM and Transformer-based architectures, demonstrate superior capabilities in capturing complex temporal dynamics, although with increased computational demands and reduced interpretability. The proposed BiLSTMTransformer model combines the strengths of both LSTM and Transformer networks, achieving a balanced performance across short and long-term forecasting tasks while maintaining scalability and robustness. This hybrid design positions it as a promising solution for applications requiring accurate and resilient time-series predictions under diverse environmental conditions.

**Table 1:** Time-series model comparison.

Model	Captures nonlinearity	Handles long-term dependencies	Handles short-term variations	Interpretability	Computational efficiency	Scalability
ARIMA	No	No	Yes	High	High	Low
ETS	Partial	Limited	Yes	High	High	Low
Linear regression	No	No	Yes	High	High	Low
GRU	Yes	Partial	Yes	Moderate	Moderate	Moderate
CNN	Yes	No	Yes	Low	Moderate	Moderate
LSTM	Yes	Yes	Yes	Low	Low	Moderate
Transformer	Yes	Yes	Partial	Low	Moderate	High
BiLSTMTransformer	Yes	Yes	Yes	Low	Moderate	High

## The BiLSTM-Transformer framework

**Fig. 1:**
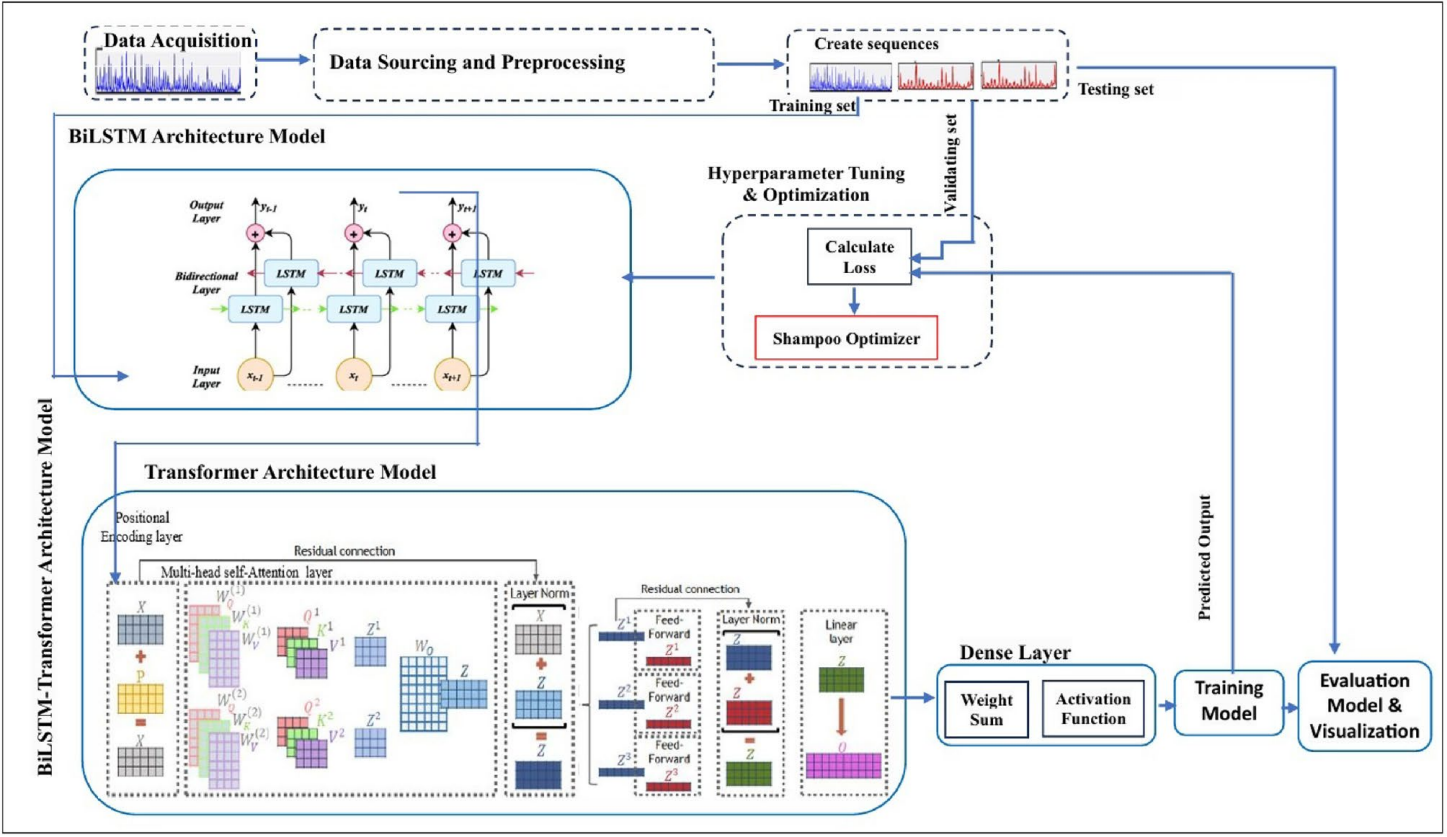
The proposed integrated BiLSTM-Transformer model.

The model introduced in this work, referred to as BiLSTM-Transformer, integrates BiLSTM with Transformer-based layers to construct a robust deep learning framework tailored for time-series prediction tasks, as illustrated in Fig. 1.

### Data sourcing and preprocessing

The dataset employed in this study was sourced from publicly accessible meteorological repositories rather than being directly recorded from sensor devices. One such source includes Open-Meteo^24^, which offers extensive environmental data. The dataset encompasses multiple key time-series variables such as solar radiation, temperature, atmospheric humidity, wind velocity, and barometric pressure, among others. Ensuring that the dataset spans a sufficient historical range and contains all necessary parameters is essential to support reliable and accurate forecasting. This dataset includes sequential temporal entries used for modeling and analysis^25^.

1 $$\begin{aligned} X=X\{x_1,x_2,...,x_T\} \end{aligned}$$

Where *T* represents the total number of time steps, and *X* denotes the unprocessed time-series data.

#### Data cleaning and outlier mitigation

The dataset is first examined to remove anomalies and any irrelevant entries that could negatively affect model training and prediction accuracy^26^.

#### Missing value treatment

Incomplete data entries are addressed using standard imputation techniques, such as forward-fill or interpolation, to maintain the sequence’s continuity^27^.

#### Normalization

To ensure consistency across features, values are scaled to a uniform range, typically [0, 1] or [-1, 1], by applying the appropriate transformation formula^28^.

2 $$\begin{aligned} {x\prime }_t=\frac{x_t-min(X)}{max\left( X\right) -min(X)} \end{aligned}$$

Where $x'_t$ denotes the normalized feature value at time *t* after applying Min-Max scaling. The original feature value before normalization is given by $x_t$, while *min*(*X*) and *max*(*X*) correspond to the minimum and maximum values within the feature set *X*, respectively. This transformation ensures all values are rescaled within a specific range, typically [0, 1], to improve training stability and performance.

#### Sequence construction

To prepare the time-series data for model training, input sequences are constructed by segmenting the data into overlapping windows. Each sequence of length *t* is used to predict the next point in the series^29^, where *n* specifies the size of the sliding window applied to generate these input-output pairs.

3 $$\begin{aligned} X_t={x_{t-n},\ x_{t-n+1},\ \ldots ,\ x_t} \end{aligned}$$

### Model architecture

This section outlines the structural design of the models implemented for handling sequence-based learning and predictive tasks.

#### BiLSTM model

The BiLSTM framework consists of two Long Short-Term Memory networks running in opposite directions^9^. One network processes the sequence from past to future, while the other traverses it from future to past. At each time step *t*, the forward LSTM computes the hidden state based on preceding inputs, enabling the model to capture temporal dependencies in a forward manner. Simultaneously, the backward LSTM computes hidden representations by moving in reverse, thereby incorporating future context into the learning process.

4 $$\begin{aligned} {\mathop {h_t}^{\rightarrow }} =LSTM\{x_t,\ h_{t-1}\} \end{aligned}$$

The notation ${\mathop {h_t}\limits ^{\rightarrow }}$ represents the hidden state at time step *t*. The LSTM derives this hidden representation by integrating information from the input at the current time step, $x_t$, along with the hidden state from the previous step, $h_{t-1}$. When the LSTM operates in the backward direction, it processes the sequence in reverse order–starting from the end and moving toward the beginning.

5 $$\begin{aligned} {\mathop {h_t}^{\leftarrow }}=LSTM \{x_t,\ h_{t+1}\} \end{aligned}$$

Here, $h_{t+1}$ refers to the hidden state corresponding to the subsequent time step, $t+1$. At time step *t*, the resulting output $h_t$ is formed by combining the hidden states from both directions into a single concatenated representation.

6 $$\begin{aligned} & h_t=[{\mathop {h_t}^{\rightarrow }},\ {\mathop {h_t}^{\leftarrow }}] \end{aligned}$$

7 $$\begin{aligned} & H_t=\{h_1,\ h_2,\ \ldots ,h_T\} \end{aligned}$$

The series of hidden states, denoted as $H_t$, is subsequently passed into the Transformer architecture.

#### Transformer architecture

The Transformer framework, as introduced in^22^, represents a significant evolution in handling sequential data. Unlike recurrent structures such as LSTMs, which process inputs step by step, the Transformer leverages a self-attention mechanism to simultaneously evaluate relationships across the entire input sequence. This allows the model to efficiently learn and represent long-range dependencies without relying on recurrence. By enabling attention over multiple parts of the input sequence at once, the Transformer achieves both scalability and effectiveness, especially when working with large datasets. This capability makes it particularly suitable for domains like time-series analysis and natural language understanding, where capturing intricate patterns across data is essential. Moreover, its ability to perform computations in parallel drastically reduces training times, contributing to its growing adoption in advanced deep learning systems. Since Transformers lack an inherent understanding of sequential order, positional encodings are integrated with the output from the BiLSTM to embed time-based contextual information into the model.

8 $$\begin{aligned} Pos\left( t,\ 2i\right) =\sin {\left( \frac{t}{10000\frac{2i}{d}}\right) \ },\ Pos\left( t,\ 2i+1\right) =\cos {\left( \frac{t}{10000\frac{2i}{d}}\right) } \end{aligned}$$

Here, *i* refers to the index of a particular feature within the vector, while *t* denotes its corresponding position within the temporal sequence. The parameter *d* signifies the total number of features, defining the model’s dimensional space. The resulting sequence after encoding can be expressed as:

9 $$\begin{aligned} z_t=h_t+{Pos}_t \end{aligned}$$

At each time step *t*, the variable $h_t$ represents the hidden state or the extracted feature vector, while $z_t$ denotes the final encoded output. The positional encoding term, ${Pos}_t$, conveys temporal information, indicating the relative or absolute position of each data point within the sequence. The Multi-Head Self-Attention mechanism is employed to determine how much focus each time step should allocate to other steps in the sequence. For every input representation $z_t$, the model performs the following computations:

10 $$\begin{aligned} Q=W_{Q^{Z_t}}\ ,\ \ \ \ \ \ K=\ \ W_{K^{Z_t}}\ ,\ \ \ \ \ \ {V=W}_{V^{Z_t}} \end{aligned}$$

In this context, *Q* denotes the query vector derived from the input representation $Z_t$ through a transformation using the weight matrix *W*, which projects $Z_t$ into the query space. Similarly, the key vector *K* is also obtained from $Z_t$, while the value vector *V* carries the core information that the model will use after applying the attention weights. The interaction between the query and key vectors is quantified by computing the attention score using the following formula:

11 $$\begin{aligned} Attention\ \left( Q,K,V\right) =softmax\ \left( \frac{QK^T}{\sqrt{d_k}}\right) V \end{aligned}$$

Here, *dk* refers to the dimensionality of the key vector. Following the self-attention computation, a Feed Forward Network (FFN) is employed to further refine and transform the resulting output.

12 $$\begin{aligned} FF\ \left( z_t\right) =RELU\left( W_1z_t+b1\right) \ W_2+b_2 \end{aligned}$$

In this context, $\text {FF}\left( z_t\right)$ indicates the output produced by the feed-forward network at time step $t$, where the input is the vector $z_t$. This input usually comes from an earlier stage in the model, such as the Transformer encoder. The transformation involves a weight matrix $W$, which projects the input into a hidden representation, and a corresponding bias term $b$ that is added during this linear transformation.

To support stable and efficient model training, the output of the feed-forward network is combined with the original input through residual connections. This combined result is then passed through a normalization layer, enabling the network to learn more effectively.

13 $$\begin{aligned} z_t^\prime =LayerNorm\left( z_t+FF\ \left( z_t\right) \right) \end{aligned}$$

The vector $z_t^\prime$ represents the processed output at time step $t$, following the operations of the feed-forward network and subsequent layer normalization.

### Dense layer

The dense layer functions as the concluding component of the model, responsible for translating the refined features extracted by the Transformer into the final prediction. It effectively transforms the high-dimensional representations generated by preceding layers into the target output format commonly a single scalar or a fixed-size vector corresponding to the forecast at a particular time step $t$. This layer acts as a bridge between the model’s internal learned representations and the actual predicted values required for evaluation or application.

14 $$\begin{aligned} \widehat{y_t}=\sigma \ \left( W_dz_t^\prime +b_d\ \right) \ \ \end{aligned}$$

At each time step $t$, the predicted output is denoted as $\widehat{y_t}$. This represents the model’s final response after processing through the transformation layers and applying a nonlinear activation function, such as ReLU or sigmoid.

### Hyperparameter tuning and optimization

Optimizing the performance of deep learning models involves systematic tuning of hyperparameters^30^. In this work, a dual-optimizer strategy combining Adam and Shampoo is employed to enhance the training efficiency and stability of the BiLSTM-Transformer architecture. Adam^31^, a widely used first-order optimizer, is effective during early training stages due to its adaptive gradient mechanisms. As training progresses, Shampoo’s second-order preconditioning capabilities become advantageous, especially for complex models like Transformers, offering more refined and stable convergence.

This hybrid approach ensures that the model benefits from Adam’s quick adaptation at the start and Shampoo’s advanced optimization in later phases. A differentiated optimization strategy is applied across the model’s layers: Adam is used for the BiLSTM layers, which are suited to sequential pattern learning, while Shampoo is allocated to the Transformer layers, which demand efficient handling of long-term dependencies. Adam dynamically adjusts learning rates using estimates of the first and second moments of the gradients, thus minimizing oscillations and promoting stable convergence. Conversely, Shampoo leverages a block diagonal approximation of the Hessian matrix to condition gradients, which proves beneficial for Transformer layers that operate in high-dimensional parameter spaces. This targeted application of optimizers ensures that each model component receives the most appropriate training dynamics based on its structural and functional needs.

### Model training

The BiLSTM-Transformer model is trained using a phased strategy that gradually transitions from Adam to Shampoo, aiming to balance rapid early learning with stable and precise later stage training. This staged training approach consists of three key phases.

In the initial phase, spanning the first 50% of the total epochs, Adam is employed exclusively across all model layers. This period focuses on rapidly stabilizing the learning process, particularly for the BiLSTM layers, by leveraging Adam’s responsive learning rate adjustments. During this stage, Shampoo is inactive to reduce computational costs.

The intermediate phase covers 50% to 75% of the training duration. Here, the model begins a gradual transition where the use of Shampoo in Transformer layers is incrementally increased. The decision to introduce Shampoo is based on signals such as plateauing validation loss or rising gradient variance. Rather than switching abruptly, a smooth blending of optimizers is performed. The influence of Adam is slowly reduced, while Shampoo’s contribution is gradually elevated. This phase uses a weighted interpolation of the two optimizers, dynamically balancing their effects on Transformer layer updates.

During the final quarter of the training epochs, the optimization process transitions such that Shampoo exclusively manages the Transformer layers, whereas Adam continues optimizing the BiLSTM components. This stage is crucial for ensuring stable convergence, as Shampoo’s second-order methods enhance the model’s ability to learn long-range dependencies typical in Transformer architectures. This adaptive optimizer handover strategy enables the model to benefit from Adam’s fast convergence during early training and Shampoo’s precision during fine-tuning. As a result, the BiLSTM-Transformer achieves improved predictive performance and enhanced generalization capability.

### Model evaluation

After training, the model’s effectiveness is assessed using several standard performance metrics. These include Mean Squared Error (MSE), Root Mean Squared Error (RMSE), Mean Absolute Error (MAE), and the coefficient of determination ($R^2$). Each metric offers a distinct perspective on how closely the model’s predictions align with the actual observed values.

15 $$\begin{aligned} & MSE=\frac{1}{n}\sum _{i=1}^{n}{{(y_t-{\bar{y}}_t)}^2\ } \end{aligned}$$

16 $$\begin{aligned} & RMSE=\sqrt{\frac{1}{n}\sum _{i=1}^{n}{(y_t-{\bar{y}}_t)}^2} \end{aligned}$$

17 $$\begin{aligned} & MAE=\ \frac{1}{n}\sum _{i=1}^{n}{|(y_t-{\bar{y}}_t)|} \end{aligned}$$

18 $$\begin{aligned} & {R}^2=1-\ \frac{\sum _{i=1}^{n}{(y_t-{\bar{y}}_t)}^2\ }{\sum _{i=1}^{n}{(y_t-\bar{y})}^2} \end{aligned}$$

Given $n$ as the total count of observations, the predicted and true values are represented by $y_t$ and $\bar{y}_t$, respectively. RMSE quantifies the square root of the average squared deviations between the actual values $\bar{y}_t$ and their predictions $y_t$. This metric is instrumental in estimating the standard deviation of prediction errors and reflects the overall accuracy of the model. Another evaluation metric, MAE, measures the mean of the absolute differences between $y_t$ and $\bar{y}_t$. $R^2$, serves as a statistical measure of how well the predictions approximate the real data distribution.

### Visualization

To monitor how the model behaves over time, a graphical comparison of predicted values $\widehat{y_t}$ against actual results $y_t$ is performed. This visual representation offers a straightforward method to assess alignment between estimated and observed data points. By studying this plot, inconsistencies can be detected, and the model’s fidelity in capturing the real-world dynamics of the dataset can be examined.

## Experimental results

The findings obtained from the experimental analysis shed light on the predictive strength of the BiLSTM-Transformer architecture in modeling meteorological behavior. Compared to standard forecasting approaches, the hybrid model demonstrates notable gains in accuracy, particularly in understanding prolonged temporal dependencies. The model’s capability to effectively synthesize five years of historical weather data and deliver dependable forecasts for the following year emphasizes its practical utility in energy planning, especially for optimizing the location of future energy-generating assets. These outcomes underscore the value of deploying advanced artificial intelligence frameworks to support data-driven decisions in the context of climate response and sustainable energy development.

### Dataset description

This study employed data provided by Open-Meteo, a freely accessible platform, to evaluate various wind speed forecasting algorithms. The datasets comprise hourly wind velocity readings recorded at two Egyptian locations: one situated at $0.0444^{\circ }$ N, $31.2357^{\circ }$ E, and the other at $31.2156^{\circ }$ N, $29.9553^{\circ }$ E. The data underwent preprocessing and were partitioned into 80% for model training and 20% for evaluation. Several predictive models were benchmarked in this study, including Linear Regression ^32^, LSTM ^33^, GRU ^34^, CNN ^35^, Transformer ^36^, and the proposed BiLSTM-Transformer framework. These models were assessed based on performance indicators such as MAE, MSE, RMSE, and $R^2$.

### Hyperparameters, model configurations, and experimental setup

This section elaborates on the design and setup of the experimental framework, including the specific models evaluated, their architectural parameters, and the selected hyperparameter values. A comprehensive summary of these configurations is provided in Table 2.

**Table 2:** Comparison of different models’ hyperparameters.

Hyperparameter	Linear regression	LSTM	GRU	CNN	Transformer	Proposed BiLSTM-Transformer
Number of layers	None (default configuration)	2	2	3	4	1
Number of epochs		20	20	20	20	20
Batch size		32	32	32	32	32
Learning rate		0.001	0.001	0.001	0.001	0.001
Layers		Hidden units per layer: 50	Number of layers: 2 hidden units per layer: 50	Number of layers: 3 convolutional layers kernel size: 3 x 3 filters per layer: 32	Number of layers: 4 number of heads: 8 hidden size: 256	LSTM layers: 1 transformer layers: 3

### Evaluation results

This study assessed the forecasting capabilities of multiple models, including CNN, Transformer, LSTM, GRU, Linear Regression, and the proposed BiLSTM-Transformer hybrid. Among these, both the LSTM and the BiLSTM-Transformer models consistently demonstrated the most accurate results across all datasets, reflected by their high $R^2$ values and minimal error metrics. The GRU model also showed competitive performance, closely aligning with LSTM in several cases. On the other hand, the CNN model underperformed, indicating limitations in handling temporal patterns effectively. Although the Transformer model is known for its strength in capturing sequential relationships, it yielded slightly higher error rates, which may be attributed to its dependence on larger volumes of data for optimal training. An overview of these results, detailing the comparative effectiveness of each model, is presented in Table 3.

**Table 3:** Model performance across different locations.

Model	Location	MAE	MSE	RMSE	$\text {R}^{2}$
Linear regression^32^	Location 1	0.2410	0.1053	0.3245	0.8906
	Location 2	0.3026	0.1991	0.4462	0.7896
LSTM^33^	Location 1	0.2077	0.0841	0.2900	0.9127
	Location 2	0.2727	0.1599	0.3999	0.8309
GRU^34^	Location 1	0.2138	0.0868	0.2946	0.9099
	Location 2	0.2692	0.1584	0.3980	0.8326
CNN^35^	Location 1	0.4102	0.2820	0.5310	0.7072
	Location 2	0.5991	0.5910	0.7687	0.3754
Transformer^36^	Location 1	0.2217	0.0967	0.3109	0.8996
	Location 2	0.2861	0.1713	0.4139	0.8189
The proposed BiLSTM-Transformer	Location 1	0.2089	0.0851	0.2917	0.9116
	Location 2	0.2779	0.1655	0.4068	0.8251

## Discussion

This study presents compelling evidence that the use of a hybrid optimization strategy significantly improves the performance of the BiLSTM-Transformer model in time-series forecasting tasks. This section explores the enhancements introduced by the proposed method compared to conventional techniques, with particular emphasis on optimization behavior, training dynamics, and predictive accuracy. The integration of two optimizers with complementary strengths allows the model to more effectively handle complex patterns by minimizing prediction errors and capturing both short-term fluctuations and extended temporal relationships.

The design incorporates adaptive learning principles that allow for swift early-stage convergence and precise parameter tuning in the later phases of training. This contributes to improved accuracy and model consistency over time. The optimizer allocation is tailored to the model’s architecture–each layer benefits from the optimizer best suited to its functional role. This targeted approach to gradient adjustment enhances training efficiency and strengthens the model’s ability to generalize across diverse datasets.

A central innovation of this framework is the deliberate, layer-specific deployment of optimizers, which supports optimal learning across the model’s components:

- BiLSTM layers: these layers are designed to capture short-term temporal dynamics typical of meteorological datasets. They are paired with an adaptive optimizer that facilitates dynamic learning rate adjustments, reducing the risk of overfitting while promoting stable learning.
- Transformer layers: responsible for modeling long-range dependencies, these layers benefit from a second-order optimization technique that leverages curvature information to guide weight updates more accurately. This results in improved convergence and better representation of extended temporal patterns.

Collectively, the model’s hybrid optimization framework presents a scalable and resilient solution for forecasting challenges in dynamic environments. By combining flexibility, precision, and computational efficiency, this approach significantly advances the capability of deep learning in handling the multifaceted nature of time-series data.

This hybrid architecture employs a targeted layer-wise approach that effectively captures both short-range and long-range dependencies, thereby maximizing the predictive capabilities of the BiLSTM-Transformer framework.

Beyond refining individual layers, the integration of the Shampoo optimizer strengthens the model’s adaptability across varying temporal scales. Renewable energy data often display irregularities, such as seasonal shifts and unpredictable weather changes. To navigate this complexity, a forecasting model must be flexible enough to respond to short-term fluctuations while maintaining accuracy over extended periods. In this architecture, the BiLSTM layers are tuned to recognize rapid changes in data, while the Transformer components provide stability in long-term pattern recognition. This balance improves prediction accuracy under dynamic and volatile weather conditions.

Evaluation across all datasets shows that the proposed BiLSTM-Transformer consistently outperforms other models, achieving the highest coefficient of determination and the lowest scores for error metrics such as MAE, MSE, and RMSE. These results establish it as the most dependable and precise option for wind speed forecasting. Accurate predictions are essential in fields like energy management, where they enhance operational planning, resource allocation, and risk mitigation strategies. The improved performance of this hybrid model makes it especially advantageous for such real-world applications.

**Fig. 2:**
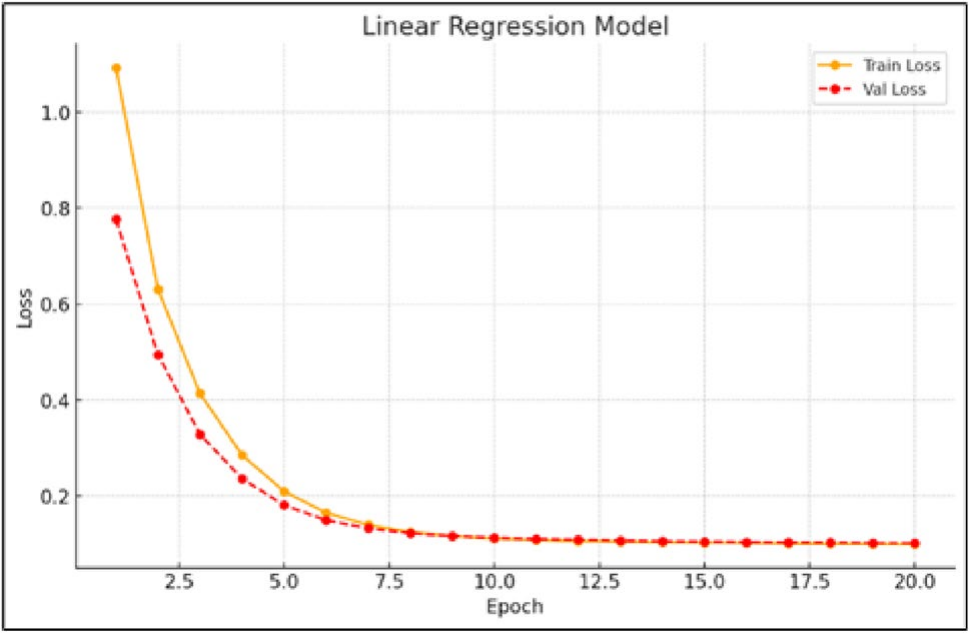
Training and validation loss curves for the linear regression model.

The study compares several models including CNN, LSTM, GRU, Linear Regression, standalone Transformer, and the BiLSTM-Transformer hybrid based on prediction accuracy and learning behavior, such as how actual and predicted wind speeds align and how their respective loss curves evolve during training. Figure 2 shows the training and validation loss curves for the linear regression model, which exhibit a steady decline. Although this indicates the model is learning, its simplistic nature limits its ability to capture the complex, non-linear dynamics of wind data. The relatively shallow loss reduction compared to advanced models highlights its inadequacy for tasks requiring nuanced temporal analysis.

**Fig. 3:**
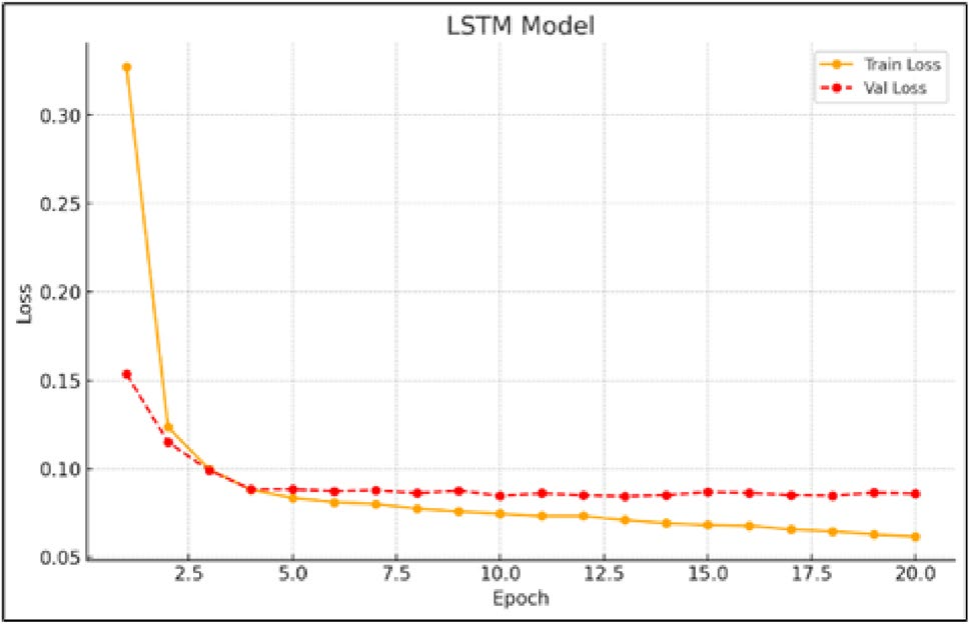
Training and validation loss curves for the LSTM model.

In contrast, Fig. 3 illustrates the strong learning capacity of the LSTM model, where the training and validation losses decrease in parallel with minimal divergence. This close alignment suggests the model generalizes well and is not overfitting. The LSTM’s ability to handle time-dependent variability confirms its suitability for wind speed prediction, particularly in scenarios where capturing temporal nuances is vital. Its robust performance demonstrates both effective pattern recognition and high accuracy in forecasting unseen data, underscoring its relevance in time-series applications.

**Fig. 4:**
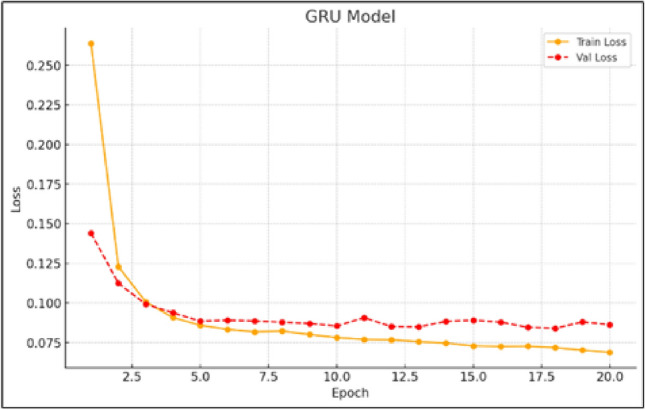
Training and validation loss curves for the GRU model.

The GRU model demonstrates strong capability in learning temporal dependencies, performing on par with the LSTM architecture, as shown by the training and validation loss patterns in Fig. 4. Although the convergence is slightly less uniform than that of LSTM, the GRU consistently achieves reductions in both training and validation errors. Its computational efficiency, paired with solid predictive accuracy, makes it a compelling alternative for wind speed forecasting tasks. While minor irregularities in the loss curves may reflect sensitivity to certain input sequences, the GRU remains effective in handling time-series data.

**Fig. 5:**
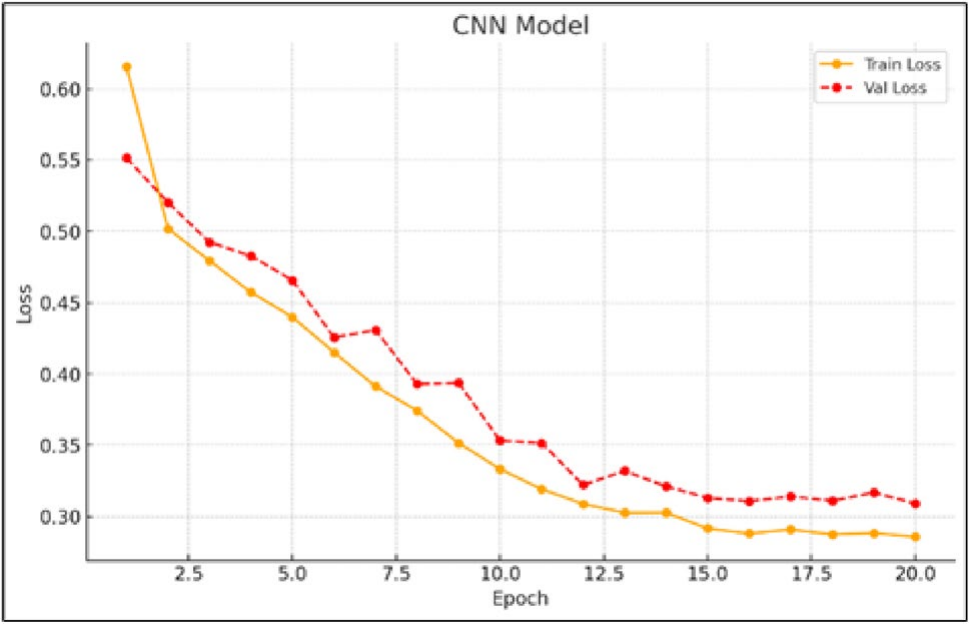
Training and validation loss curves for the CNN model.

Figure 5 presents the training and validation loss trends for the CNN model, revealing a noticeable gap between the two curves, an indication of potential overfitting and difficulty in capturing sequential patterns. Unlike GRU and LSTM, the CNN architecture shows limitations when applied to time-dependent data like wind speed. This is likely due to its inherent design, which is typically optimized for spatial feature extraction rather than temporal modeling. As a result, its performance in this context suggests that CNN alone may not be the most suitable choice for forecasting tasks that require an understanding of temporal evolution.

Nonetheless, CNN models could still offer value if used as part of a broader framework–for instance, in preprocessing stages to extract high level features or through regularization techniques to mitigate overfitting. When appropriately integrated, CNN-based components may enhance the overall learning process in hybrid models, even if they are not ideal as standalone solutions for time-series forecasting.

**Fig. 6:**
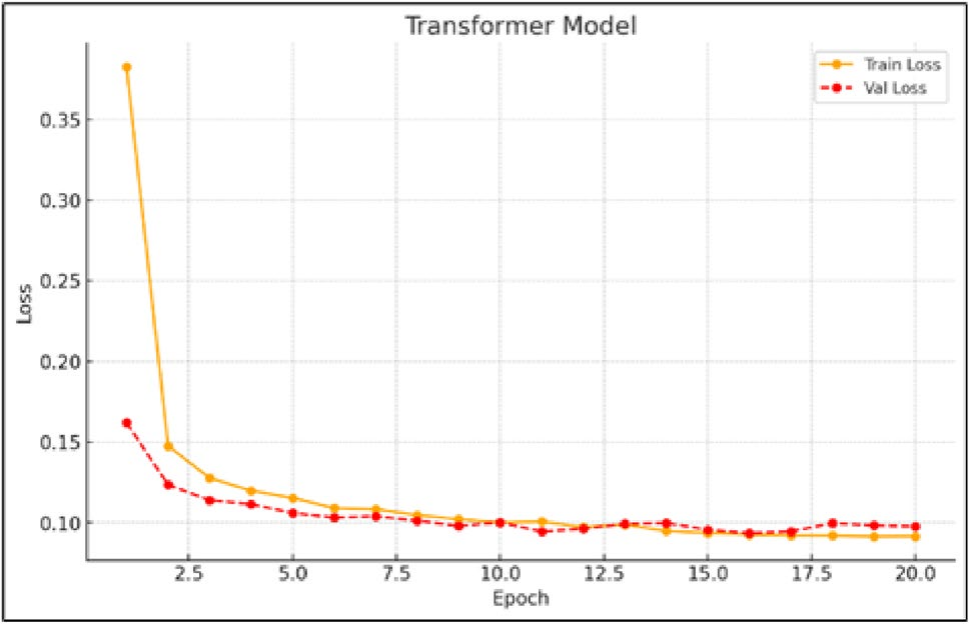
Training and validation loss curves for the transformer model.

Figure 6 illustrates the training and validation loss trajectories for the Transformer model, emphasizing its capability to handle contextual relationships and long-term dependencies within the input data. The consistent downward trend in both loss curves reflects the model’s efficient learning behavior. Its design is particularly well-suited for capturing extended temporal interactions, which contributes to its strong performance throughout training. The Transformer’s architecture proves advantageous in wind speed prediction tasks, especially when understanding broader contextual patterns is essential for accurate results.

**Fig. 7:**
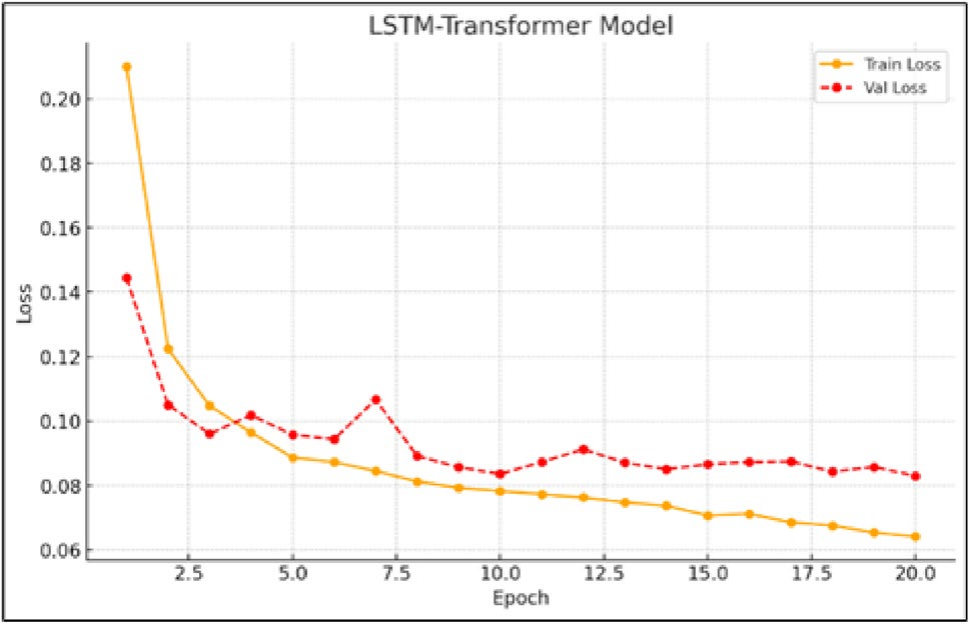
Training and validation loss curves for the proposed BiLSTM-Transformer model.

Figure 7 presents the training and validation losses for the BiLSTM-Transformer model, underscoring the effectiveness of integrating sequential memory from LSTM components with the contextual sensitivity of the Transformer. The hybrid setup achieves lower loss values while minimizing overfitting, demonstrating a balanced and robust learning process. By leveraging the complementary strengths of both architectures, this approach significantly enhances prediction accuracy. The consistently low validation loss highlights the model’s ability to identify and learn complex time-based patterns, making it a reliable tool for high-precision wind speed forecasting.

**Fig. 8:**
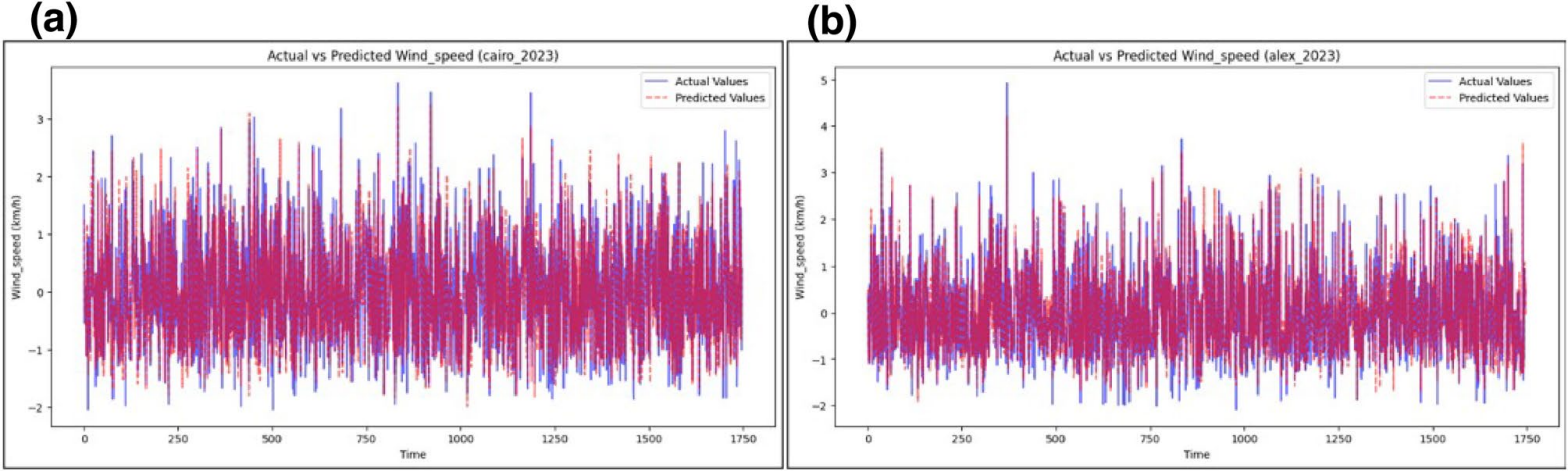
(**a,b**) Actual vs. predicted wind speed values using the proposed BiLSTM-Transformer model.

The predictive accuracy of the model is further evidenced by its performance in the Cairo 2023 and Alexandria 2023 datasets, as shown in Fig. 8a, b. The alignment between observed and predicted values remains close, even during periods of volatile weather, showcasing the model’s robustness in representing sudden changes in wind behavior. The minimal deviations observed during extreme conditions suggest that the model can maintain reliability under challenging forecasting scenarios.

**Fig. 9:**
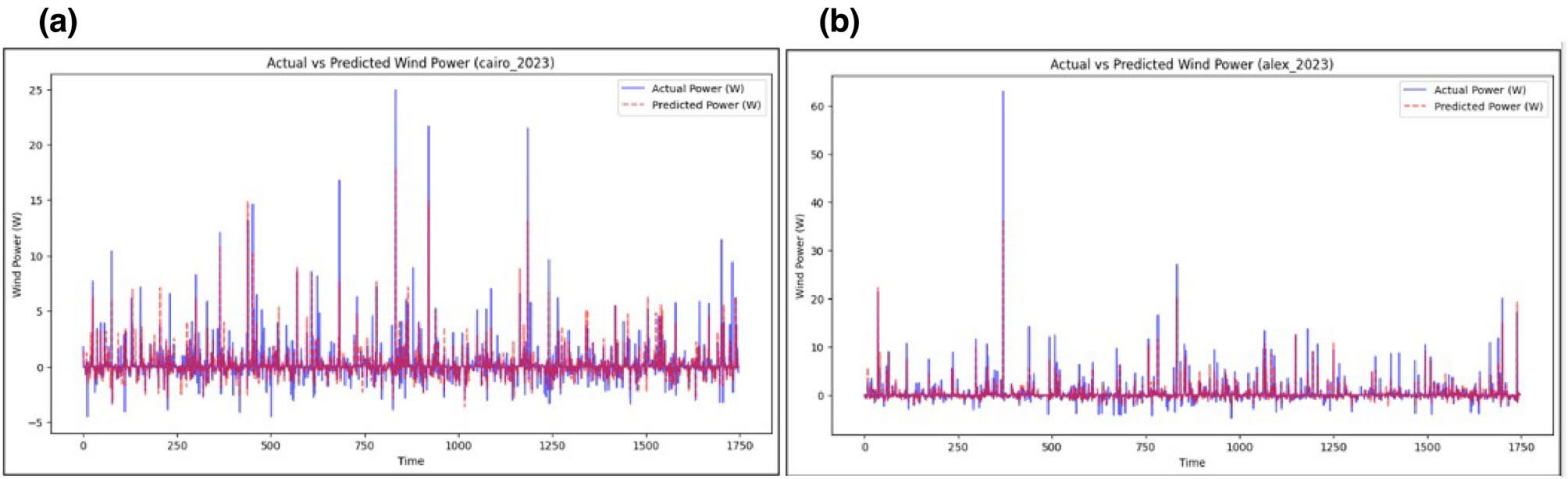
(**a,b**) Accurate wind power forecasting using the proposed BiLSTM-Transformer model.

In Fig. 9, the comparison between actual and forecasted wind power over time is shown, with solid lines representing observed values and dashed lines indicating predictions. The close overlap between the two curves highlights the model’s strong forecasting capability. It reliably captures the overall trends and peak variations in the data. Even during periods of significant fluctuation, the model maintains high alignment with real measurements, underscoring its precision and resilience in operational wind power forecasting scenarios–an essential quality for applications in sectors such as aviation, energy grid management, and renewable energy planning.

## Conclusion

This research proposes an improved optimization approach by incorporating Shampoo into the BiLSTM-Transformer architecture to increase the effectiveness of renewable energy forecasting. Shampoo, known for its advanced second-order optimization capabilities, significantly improves the model’s ability to generalize over longer training durations, resulting in more stable learning and refined predictive outcomes. By addressing the constraints of traditional single optimizer models, this approach offers more efficient training and better accuracy.

The evaluation results clearly demonstrate that the optimized BiLSTM-Transformer framework outperforms standard forecasting models. It adeptly captures both short-term temporal fluctuations through the BiLSTM layers and long-range sequence patterns via the Transformer layers. These improvements contribute to more reliable forecasts, an essential asset for managing power grids, planning infrastructure, and distributing renewable energy effectively.

From an application point of view, improved forecast accuracy can lead to more informed decision making in energy planning, resource allocation, and cost management. The method is especially relevant in contexts where renewable energy sources must be integrated into existing systems. However, the increased computational demands associated with Shampoo remain a key consideration in scaling up the model. Future work may explore adaptive learning strategies, support for real-time prediction, and application to multiple types of renewable energy data.

In summary, this study emphasizes the potential for advanced optimization in AI-powered forecasting and its role in the development of intelligent, sustainable energy solutions. The proposed framework bridges the gap between model sophistication and practical usability, offering valuable contributions to the evolution of smart energy systems.

## Data Availability

This study uses data from the Open-Meteo historical weather API, including hourly weather data for five cities in Egypt from 2020-2024, for further inquiries. This dataset can be accessed through the Open-Meteo website at https://open-meteo.com.
